# Causal relationship between gut microbiota and hidradenitis suppurativa: a two-sample Mendelian randomization study

**DOI:** 10.3389/fmicb.2024.1302822

**Published:** 2024-01-29

**Authors:** Chengling Liu, Xingchen Liu, Xin Li

**Affiliations:** ^1^Center of Burns and Plastic Surgery and Dermatology, The 924th Hospital of Joint Logistics Support Force of the PLA, Guilin, China; ^2^Department of Pathology, Changhai Hospital, Naval Medical University, Shanghai, China

**Keywords:** hidradenitis suppurativa, gut microbiota, Mendelian randomization, gut-skin axis, genome-wide association study

## Abstract

**Background:**

Accumulating evidence suggests that alterations in gut microbiota composition are associated with the hidradenitis suppurativa (HS). However, the causal association between gut microbiota and HS remain undetermined.

**Methods:**

We performed a bidirectional two-sample Mendelian randomization (MR) analysis using genome-wide association study summary data of gut microbiota and hidradenitis suppurativa from the MiBioGen consortium which concluded 18,340 individuals analyzed by the MiBioGen Consortium, comprising 211 gut microbiota. HS data were acquired from strictly defined HS data collected by FinnGenbiobank analysis, which included 211,548 European ancestors (409 HS patients, 211,139 controls). The inverse variance weighted method (IVW), weighted median (WME), simple model, weighted model, weighted median, and MR-Egger were used to determine the changes of HS pathogenic bacterial taxa, followed by sensitivity analysis including horizontal pleiotropy analysis. The MR Steiger test evaluated the strength of a causal association and the leave-one-out method assessed the reliability of the results. Additionally, a reverse MR analysis was carried out to seek for possible reverse causality.

**Results:**

By combining the findings of all the MR steps, we identified four causal bacterial taxa, namely, Family XI, Porphyromonadaceae, *Clostridium innocuum* group and Lachnospira. The risk of HS might be positively associated with a high relative abundance of *Clostridium innocuum* group (Odds ratio, OR 2.17, *p* = 0.00038) and Lachnospira (OR 2.45, *p* = 0.017) but negatively associated with Family XI (OR 0.67, *p* = 0.049) and Porphyromonadaceae (OR 0.29, *p* = 0.014). There were no noticeable outliers, horizontal pleiotropy, or heterogeneity. Furthermore, there was no proof of reverse causation found in the reverse MR study.

**Conclusion:**

This study indicates that *Clostridium innocuum* group and Lachnospira might have anti-protective effect on HS, whereas Family XI and Porphyromonadaceae might have a protective effect on HS. Our study reveals that there exists a beneficial or detrimental causal effect of gut microbiota composition on HS and offers potentially beneficial methods for therapy and avoidance of HS.

## 1 Introduction

Hidradenitis suppurativa (HS), also known as acne inversa, is a chronic, persistent, inflammatory skin condition brought on by obstruction of the infundibular portion of the pilosebaceous unit (Rathod et al., 2023). It often misinterprets as an infection, with the high impact on the patient’s quality of life among all the assessed dermatological diseases. The global epidemiological survey indicates that HS prevalence varies from 0.03 to 4% (Calao et al., 2018). The pathophysiology and etiology of HS are yet undetermined. However, several variables are thought to be linked to the onset and worsening of HS, including as inflammation, genetics, microbiome, environmental components, age, BMI, smoking status, work status, and income (Nguyen et al., 2021).

Recent research has also revealed an underlying gut-skin axis, where the gut and immune system are in close communication and collaborate to guard against external antigens (De Pessemier et al., 2021; Sinha et al., 2021; Mahmud et al., 2022). Utilizing antibiotics with extra anti-inflammatory properties is one of the treatment strategies for HS, while it is yet unknown how their antimicrobial action directly affects the microbiota (Frew et al., 2019). Multiple studies have shown a connection between changes in intestinal flora abundance and the onset and progression of HS, albeit the nature of this connection is still unidentified (Ellis et al., 2019). For instance, in HS patients, research found that Bilophila species were more prevalent while Lachnobacterium species were less prevalent (Kam et al., 2021). Further research found that the increased inflammatory markers, such as TNF-α, IL-6, IL-8, and C-reactive protein, were linked to lower microbial diversity and increased sluggishness and deteriorating wellness metrics which might be related to the HS (Öğüt et al., 2022). Yet, there was occasionally disagreement in the epidemiological data supporting these relationships, which might be caused by different measuring techniques, selective bias and confounding factors. As an instance, it had been found that Firmicutes levels were either greater (Haskin et al., 2016) or lower (Kam et al., 2021) in HS patients compared to healthy controls.

Although clinical data validates this link, common cofounders, selective bias (such as age, gender, and area), and variability in investigations tend to render it tough to comprehend. So as to better understand the processes behind HS and supply evidence-based strategies for clinical counseling, it may be desirable to investigate the relationship between the gut microbiota and HS as well as to further examine a causative correlation.

Based on a genetic standpoint, Mendelian randomization (MR) examines potential causal relationships between exposures and outcomes through applying the genetic law of random distribution of gamete alleles (Richmond and Davey, 2022; Sanderson et al., 2022). The genetic variations are limited to influencing the exposure components in order to impact the outcome indicators, which can lower the interference of confounding and reverse causal relationships. MR requires a genetic variant that is strongly related with exposure as an instrumental variable (Lousdal, 2018). Using the abundance of the gut microbiota as the exposure factor and the occurrence of HS as the outcome, the two-sample Mendelian randomization method was used in this study to analyze the potential causal relationship between the gut microbiota and HS while examining their genetic relationship (Sekula et al., 2016; Bowden and Holmes, 2019).

In the present research, we used a Mendelian randomization methodology to assess a deeper connection between the gut responsible microbiota and HS. In addition to providing possible targets for therapy for the medical treatment of psoriasis, it was projected that our results might assist in the study of the inflammatory conditions underlying HS.

## 2 Methods

### 2.1 Ethics statement and overall study design

**Figure 1:**
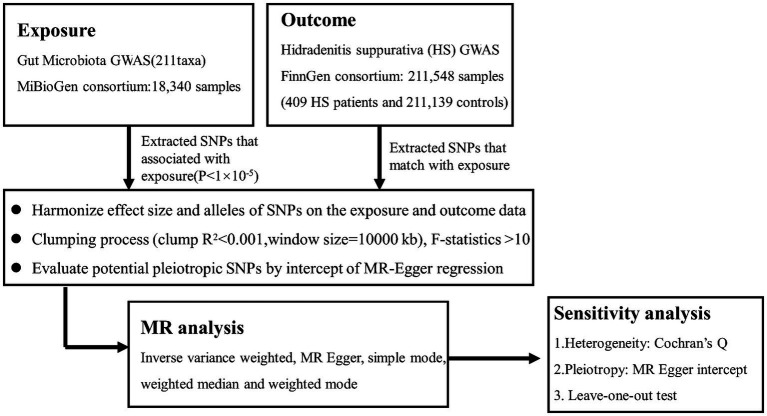
Flowchart of this Mendelian randomization study. MR. Mendelian randomization; HS. Hidradenitis suppurativa.

Our analysis used the summary statistics of publicly available GWASs. No new data was collected, and no new ethical approval was conducted. The whole process that we studied was presented in the flow chat in Figure 1. Concisely, we conducted a two-sample MR study to evaluate the causal effect between gut microbiota and HS (Jia et al., 2019). The validity of MR is based on three main assumptions (Bowden et al., 2015): (1) relevance--the relationship between genetic variants and exposure was robust; (2) independence the genetic variants were independent of confounding factors affecting exposure and outcome; and (3) exclusion restriction-the genetic variants influenced the risk of the outcome through exposure rather than other potential pathways. We used a two-sample MR computational model to investigate if there were directional causal relationships between gut microbiota and HS (the random-effects inverse variance weighted (IVW), MR-Egger regression, weighted median, reverse MR, and MR Steiger). Finally, several sensitivity analyses (the heterogeneity test, the pleiotropy test, and leave-one-out) were conducted sequentially.

### 2.2 Data sources

The GWAS summary statistics used in our study were presented in Table 1. The individuals from the data sources were from European ancestry mostly.

**Table 1:** Summary of genome-wide association studies (GWAS) datasets.

Trait	Year	Population	Sources	Sample sizes	cases	control
Exposure						
GM taxa	2021	72.3% European	MiBioGen	18,340	-	-
Outcome						
HS	2021	European	FinnGen	211,548	409	211,139

GM, gut microbiota; HS, hidradenitis suppurativa.

#### 2.2.1 Gut microbiota

GWAS data for intestinal bacteria were extracted from the MiBioGen consortium^1^, which gathered whole genome genotyping data from 18,340 participants as long as the 16S rRNA genes from participant’s fecal microbiomes (Kurilshikov et al., 2021). The 20 cohort studies included single ancestry samples from various regions, such as European (*n* = 13,266), Middle Eastern (*n* = 481), Latin American (*n* = 1,097), East Asian (*n* = 811), African American (*n* = 114). The data collected from four cohorts included multiple ancestries (*n* = 2,571). Three different variable regions (V1-V2, V3-V4, and V4) of the 16S rRNA gene were targeted in order to profile the microbial composition. A total of 211 taxa (131 genera, 35 families, 20 orders, 16 classes, and 9 phyla) were included. In our study, we finally included 194 taxa (119 genera30 families, 20 orders, 16 classes, and 9 phyla) after excluding unknown gut microbes.

#### 2.2.2 HS data sources

Summary statistics data for HS in individuals of European ancestry were obtained from the publicly available GWAS analyses. The study investigated HS cases (409) and controls (211,139), which included over 16 million genetic variants (Bao et al., 2023).^2^

### 2.3 Selection of genetic instrumental variables

We selected single-nucleotide polymorphisms (SNPs) associated with gut microbiota with a relatively tolerant significance level (*p* < 1.0 × 10^−5^) (ensuring sufficient variables for screening) (Din et al., 2019). Then, we applied chain disequilibrium *r*^2^ < 0.001 within the distance of 10,000 kb, as a cutoff of linkage disequilibrium, for respective independence before being used as primary genetic instruments. After coordinating with responsive results, every pair of combinations was retrieved for additional analysis. To detect bias from weak instrumental factors, the F statistic of IVs was determined (*F* = *R*^2^(n-k-1)/k(1-R^2^)) (n is the sample size, k is the number of included instrumental variables (IVs), and *R*^2^ is the exposure variance explained by the selected SNPs). An F statistic of more than 10 is suggestive of a strong instrument. Finally, a reverse MR study was performed to investigate the reverse causal relationship, and a nominal causal effect was defined as one with a *p*-value between 0.05.

### 2.4 Statistical analysis

The inverse variance weighted (IVW fixed model) method was employed as the main analysis, to obtain an unbiased estimate of the causal relationship between gut microbiota and HS. To evaluate the effect sizes of causation, odds ratios (ORs) of the exponential type and related confidence intervals (CIs) were mostly used and *p* value <0.05 was considered statically significant. Furthermore, the weighted median, MR Egger, simple mode, and weighted mode methods were applied as additional methods to estimate causal effects under different conditions. If at least half of the weight was generated from accurate IVs, the weighted median technique might incorporate data on many genetic variants into a single causal estimate and produce a consistent estimate (Bowden et al., 2016). The MR-Egger technique and MR-PRESSO (MR pleiotropy residual sum and outlie) might quantify the causal influence and determine whether genetic variations exhibit directional pleiotropy (Li and Li, 2023). In order to evaluate horizontal pleiotropy, the intercept of MR-Egger regression and MR-PRESSO were computed. The *p* value of >0.05 suggested that there was a low likelihood that pleiotropy would have an impact on the causal analysis. The IVW estimation method was used to produce Cochran’s Q test, which was utilized to find heterogeneity among instrumental variables. In order to determine horizontal pleiotropy and eliminate probable outliers, we also used the Mendelian randomization pleiotropy residual sum approach. To assess the level of influence of a single SNP’s causal association effect, the leave-one-out strategy, which systematically deleted one of the SNPs and utilized the remaining SNPs as instrumental variables for two-sample MR analysis, was applied (Dong et al., 2023). Moreover, MR Steiger directionality test was conducted to comprehensively assess the association between exposure and outcomes. The MR Steiger process made the assumption that a suitable genetic variation should explain more variance during exposure than during outcome. This strategy assured that the genetic instruments meet the requirements for a legitimate MR inquiry and aid in the identification of potential bidirectional effects (Hemani et al., 2017). Finally, we carried out reverse MR analysis to examine the effect of hidradenitis suppurativa on the identified gut microbiota. SNPs related to hidradenitis suppurativa were used as IVs.

If the following conditions were satisfied, we thought there was a significant causal link between systemic gut microbiota and HS: (1) The IVW method showed a significant difference (*p* < 0.05); (2) the five methods yielded consistent estimates; (3) the Cochran’s Q test, MR-Egger and MR-PRESSO were not significant (*p* > 0.05); and (4) the value of MR Steiger directionality <0.05. The ‘TwoSampleMR’ package in R software (version 4.2.2) were used for all MR analyses.

## 3 Results

### 3.1 Selection of instrumental variables

After screening at a relatively loose threshold (*p* < 1 × 10^−5^) and LD clumping, SNPs of gut microbiota ranging from phylum to genus levels were applied (Supplementary Table S1). The detailed information of the final SNPs for each bacterial trait were shown in (Supplementary Table S2). All the F statistics of the instrumental variables were over 10, implying that there was no weak instrument bias.

### 3.2 Causal impact of gut microbiota on HS

**Figure 2:**
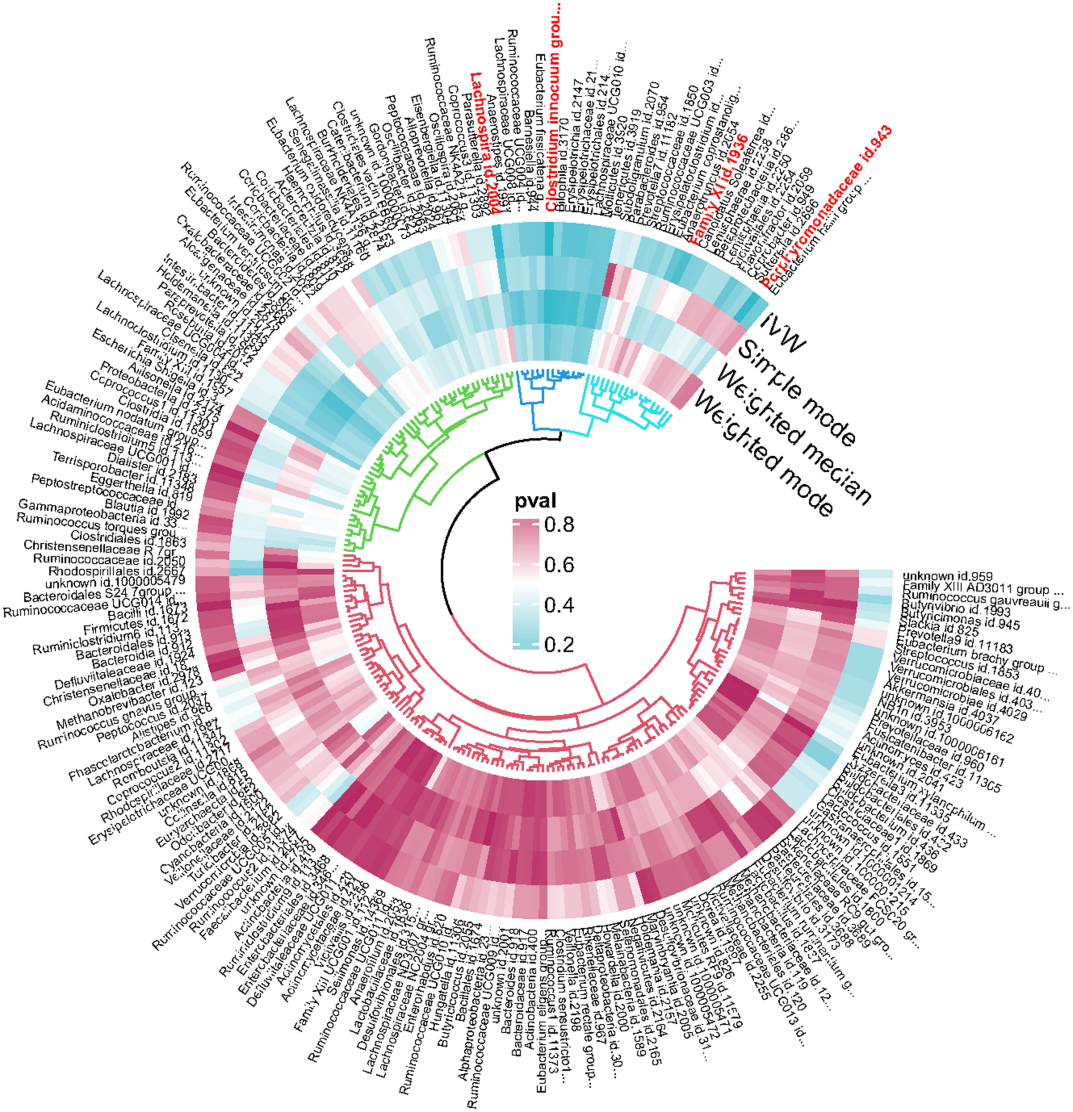
Overview of the causal role of gut microbiota in hidradenitis suppurativa. The Cyan Process color indicates statistical significance (*p* < 0.05). IVW inverse variance weighted method.

An overview of the causal effect of 211 gut microbiota taxa on HS is shown in Figure 2. Of all the genera, four significant bacterial genera were selected for further MR analyses. Furthermore, eight independent SNPs were associated with Family XI, nine independent SNPs were associated with Porphyromonadaceae and *Clostridium innocuum* group, respectively. Fifteen independent SNPs were associated with Lachnospira. SNP detailed message (Position, SD, R^2^, F) of significant genera in MR analyses were shown in Table 2.

**Table 2:** SNP message of significant genera.

Bacterial taxa	SNP	Position	SD	R^2^	F
Family XI	rs488164	chr1:239995451	3.05191	0.001493	21.38573
	rs3733511	chr4:119955787	3.285839	0.001521	21.7952
	rs11547158	chr7:148921732	4.458915	0.001584	22.69556
	rs10759623	chr9:115821485	3.845507	0.001774	25.42479
	rs2155352	chr11:95357209	3.615968	0.00173	24.79485
	rs697771	chr16:54115200	3.008026	0.001528	21.89466
	rs2156611	chr18:43345864	3.003337	0.001401	20.06813
	rs6025153	chr20:55348292	3.133351	0.001431	20.50259
Porphyromonadaceae	rs35961441	chr1:240929774	2.479539221	0.00136086	19.49226802
	rs17065783	chr3:62035586	1.462024624	0.001633433	23.40284583
	rs3111851	chr3:189854046	1.449011307	0.001610304	23.07094669
	rs864093	chr4:149825977	1.405378421	0.001409383	20.18827212
	rs6953849	chr7:69251692	1.802774978	0.001584594	22.70200933
	rs10858364	chr9:138076081	1.445458959	0.001462565	20.95116738
	rs10119172	chr9:2233969	1.705486081	0.001865821	26.73859622
	rs10762312	chr10:71571863	1.421106832	0.001356281	19.42659748
	rs7330827	chr13:23531802	2.838948375	0.001336322	19.14032548
*Clostridium innocuum* group	rs6577484	chr1:8419420	4.316127	0.00138	19.76395
	rs1948423	chr3:103025517	2.80181	0.001507	21.59581
	rs40656	chr5:9368158	3.72005	0.001469	21.04018
	rs6890185	chr5:71186626	2.788498	0.001652	23.66941
	rs4869133	chr5:95717619	4.897995	0.001358	19.44795
	rs10074000	chr5:113126539	2.721171	0.001421	20.35671
	rs71564433	chr6:143718234	3.285109	0.001487	21.29547
	rs10506058	chr12:30268485	2.654406	0.001409	20.1844
	rs77845139	chr15:59704208	3.076142	0.001396	19.99163
Lachnospira	rs4649206	chr1:24525438	1.372809246	0.001161701	16.63630035
	rs10910677	chr1:144957815	1.662750253	0.001231006	17.63000724
	rs2249707	chr1:159922959	1.423379379	0.001249964	17.90186416
	rs12127606	chr1:245812704	2.738143012	0.001074421	15.38504135
	rs6663859	chr1:220543773	1.322585976	0.001148805	16.45140656
	rs1929912	chr1:195644726	1.430543879	0.001219812	17.46949656
	rs511419	chr1:106598234	2.595558682	0.001034988	14.81979988
	rs863578	chr1:172693116	1.356303385	0.001178382	16.87546345
	rs1326854	chr1:193714589	1.490826156	0.001198946	17.17030294
	rs4908554	chr1:6568387	1.371732777	0.001093848	15.66354236
	rs10926758	chr1:242674732	1.366362392	0.001317977	18.87721724
	rs56791201	chr2:57701660	1.324021269	0.001529857	21.91661095
	rs77041968	chr2:106243104	1.933709518	0.001279022	18.3185652
	rs10928512	chr2:135451302	1.362439259	0.001141828	16.35138024
	rs10932829	chr2:220702824	1.446989937	0.001245704	17.84077438

SD, standard deviation; SNP, single nucleotide polymorphism.

**Figure 3:**
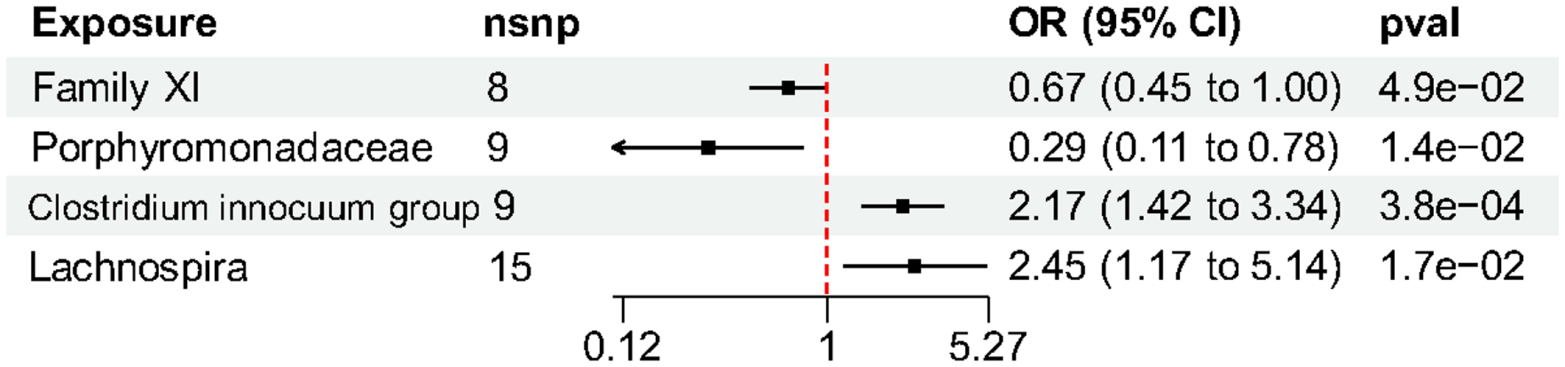
Forest plot of gut microbiota taxa associated with hidradenitis suppurativa identified by the inverse variance weighted method.

At the family level, we found that Family XI (OR 0.67, 95% CI 0.45–1.00, *p* = 0.049) and Porphyromonadaceae (OR 0.29, 95% CI 0.11–0.78, *p* = 0.014) had a nominally protective role in HS by the primary IVW method (Table 3). For another, at genus level *Clostridium innocuum* group (OR 2.17, 95% CI 1.42–3.34, *p* = 0.00038) and Lachnospira (OR 2.45, 95% CI 1.17–5.14, *p* = 0.017) had an increased risk of developing HS (Figure 3). All the MR Steiger directionality tests indicated a consistent trend from gut microbiota to HS for all outcomes.

**Table 3:** Significant MR analysis results.

Bacterial taxa (exposure)	MR method	No. SNP	OR	95% CI	*p* value	*p* for MR-PRESSO global test
Family XI	IVW	8	0.67	0.45–1.00	0.0495	
	Weighted median		0.75	0.45–1.24	0.2622	
	MR-Egger		1.45	0.11–18.8	0.7857	
	Simple mode		0.76	0.35–1.63	0.5035	
	Weighted mode		0.77	0.37–1.60	0.5075	
	MR-PRESSO					0.934
Porphyromonadaceae	IVW	9	0.29	0.11–0.78	0.0136	
	Weighted median		0.47	0.12–1.81	0.2726	
	MR-Egger		4.64	0.06–349.81	0.5090	
	Simple mode		0.63	0.07–5.91	0.6935	
	Weighted mode		0.78	0.11–5.58	0.8123	
	MR-PRESSO					0.631
*Clostridium innocuum* group	IVW	9	2.17	1.41–3.34	0.00038	
	Weighted median		2.06	1.16–3.67	0.0135	
	MR-Egger		2.44	0.31–19.36	0.4271	
	Simple mode		2.25	0.93–5.45	0.1093	
	Weighted mode		2.21	0.98–4.98	0.0922	
	MR-PRESSO					0.401
Lachnospira	IVW	15	2.45	1.17–5.14	0.0174	
	Weighted median		1.98	0.72–5.42	0.1832	
	MR-Egger		2.80	0.06–141.65	0.6158	
	Simple mode		5.50	0.98–31.01	0.0737	
	Weighted mode		1.43	0.29–7.11	0.6691	
	MR-PRESSO					0.729

OR, odds ratio; CI, confidence interval; SNP, single nucleotide polymorphism; MR, mendelian randomization; MR-PRESSO (MR pleiotropy residual sum and outlie).

### 3.3 Sensitivity analysis

**Figure 4:**
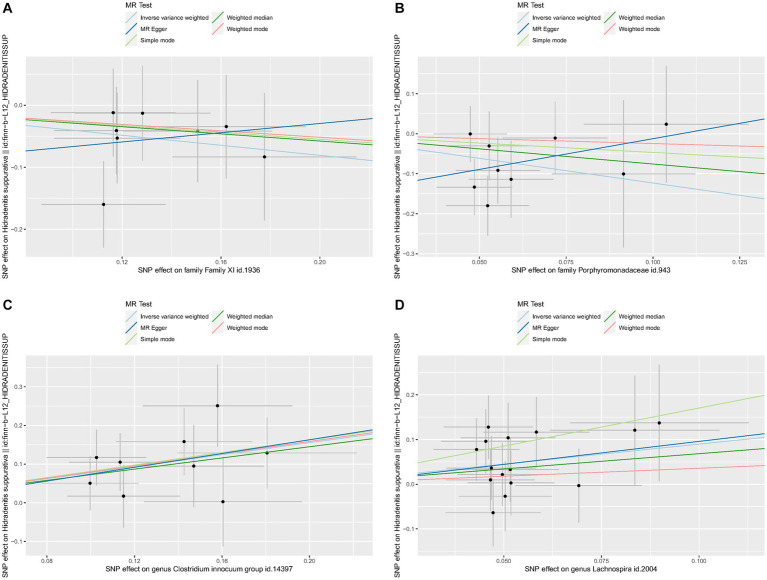
Scatter plots of each genus associated with the risk of hidradenitis suppurativa. **(A)** Family XI, **(B)** Porphyromonadaceae, **(C)** *Clostridium innocuum* group, **(D)** Lachnospira.

**Figure 5:**
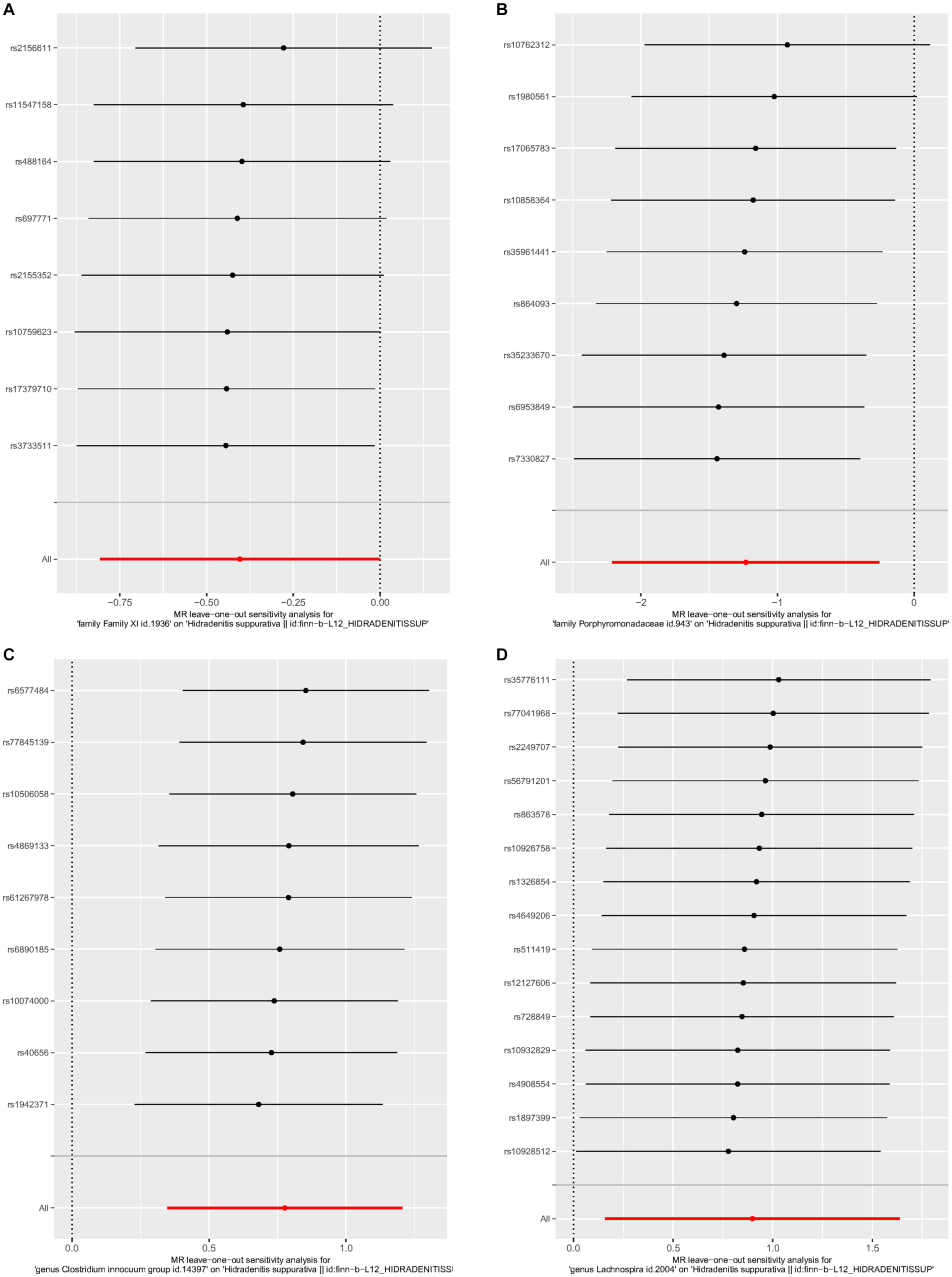
Leave-one-out analysis of each genus associated with hidradenitis suppurativa. **(A)** Family XI, **(B)** Porphyromonadaceae, **(C)** *Clostridium innocuum* group, **(D)** Lachnospira.

**Figure 6:**
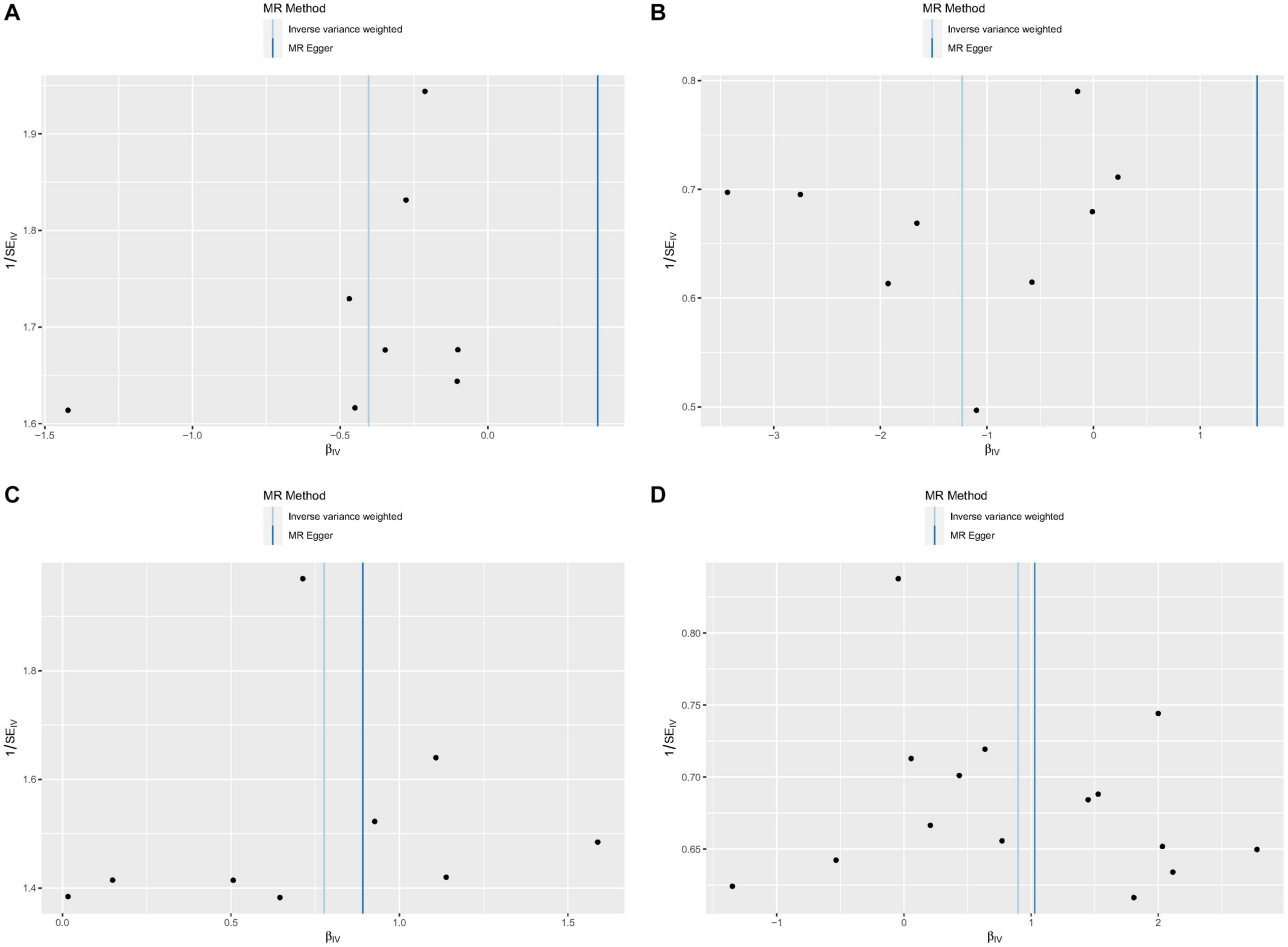
Funnel plots of each genus associated with hidradenitis suppurativa. **(A)** Family XI, **(B)** Porphyromonadaceae, **(C)** *Clostridium innocuum* group, **(D)** Lachnospira.

No heterogeneity was found within the IVs of all the four genera by Cochrane’s Q test (Table 4). The MR-Egger regression intercepts and the MR-PRESSO indicated no horizontal pleiotropy and outlier values (*p* > 0.05). The scatter plots illustrated that Family XI and Porphyromonadaceae might have protective effect on HS, meanwhile, *Clostridium innocuum* and Lachnospira might have anti-protective effect on HS. The IVW method, MR-Egger, weighted median, weighted mode, and simple mode are the methods of MR analysis that have weights and were described in the scatter plots. Positive markers of the association between the genus and HS were discovered to be the lines sloping upward from left to right, whereas protective genera were found to be those sliding downward from left to right (Figure 4). There were no potential outliers of the IVs of all four genera for HS in “leave-one-out” analysis (Figure 5), implying that all the identified causal associations were not influenced by single IV. Furthermore, the funnel plots showed no observable horizontal pleiotropy for any outcome (Figure 6). Analysis of the reverse MR data showed that HS had no causal effect on the screened gut microbiota (Table 5).

**Table 4:** Sensitivity analysis of 4 taxa associated with HS.

Exposure	SNPs	MR-Egger intercept		Cochrane’s Q IVW		Cochrane’s Q Egger		Correct causal direction
		Intercept value	*p*-value	Q value	*p*-value	Q value	*p*-value	
Family XI	8	−0.104	0.573	3.42	0.844	3.06	0.802	True
Porphyromonadaceae	9	−0.166	0.239	6.40	0.602	4.75	0.691	True
*Clostridium innocuum* group	9	−0.015	0.915	4.16	0.843	4.14	0.763	True
Lachnospira	15	−0.007	0.948	8.11	0.884	8.10	0.837	True

SNPs, single-nucleotide polymorphism; IVW, inverse-variance weighted; MR, Mendelian randomization; HS hidradenitis suppurativa.

**Table 5:** Reverse MR results of causal links between HS and gut microbiota.

Bacterial taxa (outcome)	MR method	No. SNP	OR	95% CI	*p*-value
Family XI	IVW	5	1.01	0.94–1.08	0.798
	Weighted median		1.04	0.96–1.08	0.278
	MR-Egger		1.17	0.99–1.40	0.278
Porphyromonadaceae	IVW	5	1.02	0.97–1.05	0.73
	Weighted median		1.00	0.97–1.05	0.89
	MR-Egger		1.01	0.89–1.16	0.82
*Clostridium innocuum* group	IVW	5	0.98	0.93–1.03	0.37
	Weighted median		0.97	0.90–1.03	0.31
	MR-Egger		0.92	0.78–1.09	0.41
Lachnospira	IVW	5	0.99	0.95–1.02	0.42
	Weighted median		0.98	0.94–1.02	0.30
	MR-Egger		0.88	0.76–1.03	0.25

## 4 Discussion

Firmicutes, Bacterioidetes, Actinobacteria, and Proteobacteria constitute the majority of normal gut microbiota (Liu et al., 2023). These organisms are largely steady and unchanging at the phylum level but vary greatly at the species level (Christovich and Luo, 2022). In conjunction with immunological modulation, the gut microbiota serves as essential underlying metabolism and intestinal permeability equilibrium (Qiu et al., 2022; McCallum and Tropini, 2023). According to epidemiological data, there was a link between HS disease development and gut microbiota. Our current study comprehensively examined the link of causality between gut microbiota and HS using pooled GWAS data. To the best of our knowledge, this study is the first to use MR method to investigate the bidirectional causative relationship between gut microbiota and HS. With the help of extensive GWAS summary statistics, we thoroughly assessed the causal impact of 211 GM taxa (from phylum to genus level) on HS in this investigation. Finally, we revealed several types of microbes in the gut that are formally linked to HS. Family XI (a family in Clostridiales also known as Clostridium cluster XI) and Porphyromonadaceae were identified to possess a role of prevention in HS, however the *Clostridium innocuum* group and Lachnospira were ostensibly linked to higher risk for HS. By using pleiotropy analysis, no significant pleiotropic variant among the chosen genetic instrumental variants was discovered in the datasets. Notably, results from five MR analysis techniques showed that the specific microbiome instrumental variations strongly affected the risk of HS not via other mechanisms. These findings suggest a causal relationship between HS and gut microbiome.

It is assumed that HS has a complex pathophysiology, with factors including microbiome, environment, lifestyle, and genetics all playing a role. There is a growing mount of knowledge on HS that suggests pathogenic bacteria may play a part and mounting evidence that the development and maintenance of host homeostasis depend on gut microbiota having constant communication with one another (Alves et al., 2022). Unfortunately, a complete understanding of the precise mechanism underpinning gut-skin microbial interactions is still lacking. McCarthy and Kam found that HS patients exhibited lower abundances of beneficial Lachnobacterium and Veillonella and higher levels of *Ruminococcus gnavus*, *Clostridium ramosum*, Bilophila, and Holdemania when compared to the healthy control group (Kam et al., 2021; McCarthy et al., 2022). Increased amounts of the bacterial metabolite Trimethylamine oxide (TMAO) in the bloodstream were discovered by Barrea in HS patients, and these levels were found to be correlated with higher HS Sartorius scores (Barrea et al., 2021). Luck et al. (2022) speculated that in addition to microbial dysbiosis, bacterial processes producing toxic compounds might also be linked to or involved in the development of HS.

In our study, one of the guarded microbiota (Porphyromonadaceae and Family XI) found come from the phylum Bacteroidetes and Clostridiales, which were capable of producing short-chain fatty acids (SCFAs) and were thought to preserve intestinal barrier function by preventing the passage of proinflammatory molecules into the systemic circulation and preventing the occurrence of metabolic endotoxemia (Brahe et al., 2013). There was evidence linking higher levels of acetate, n-Butyrate, and propionate to a higher Porphyromonadaceae abundance (Kelder et al., 2014). Through the production of short-chain fatty acids, Porphyromonadaceae taxa might likely play a role as adiposity modulators (Peng et al., 2009). To fulfill its function of safeguarding the gut and the human body, Jennings et al. discovered that individuals with higher relative abundances of Porphyromonadaceae had lower levels of adipose tissue and systemic inflammation (Jennings et al., 2023). Moreover, Valkonen et al. observed that Family XI could provide protection against allergic illness by contrasting the intestinal flora of the healthy control group with that of allergic disease patients (Valkonen et al., 2015). Previous research investigations had also noted that patients with HS showed a reduction in SCFAs and bacteria that produced SCFAs, such as Veillonella and Prevotella, which was consistent with our findings (Kam et al., 2021; Jiminez and Yusuf, 2023). At the same time, Tatian et al. found that HS individuals who received the adalimumab treatment showed a shift in the composition and function of the gut microbiota with significantly increased SCFA acetate (Tatian et al., 2022). The primary byproducts of the gut microbial fermentation of dietary fiber were SCFAs, which also included butyrate and propionate (Kam et al., 2021). By lowering the synthesis of pro-inflammatory cytokines including IL-1β, IL-6, IL-17, TNF-α and Th17 as well as immune cell proliferation, SCFAs and butyrate exhibited anti-inflammatory properties (Vinolo et al., 2011). Additionally, the intestinal dysbiosis and this increased cytokine outputs were what caused the HS to emerge (Molnar et al., 2020). More precisely, butyrate could stimulate Foxp3 enhancer and suppress histone deacetylase (HDAC). The host was thus protected against inflammation by peripherally derived Treg cells, which were stimulated to develop into naive CD4 + T cells when Foxp3 became expressed (Scheinfeld, 2013; Singh et al., 2014; Li et al., 2021). It suggested that the loss of butyrate-producing bacteria might be a major factor in the pathophysiology of HS by promoting a local inflammatory response, which weakened the gut epithelial barrier’s ability to regulate the presentation of gastrointestinal antigens to immune cells and systemic circulation (Bischoff et al., 2014).

*Clostridium innocuum* was a gram-positive, spore-forming, anaerobic bacterium (Cherny et al., 2021). According to recent research, it had been linked to diarrhea caused by antibiotics that resembled *Clostridium difficile* and extraintestinal infections (Chia et al., 2018). Despite the fact that no direct research had explored at the pathophysiology of *Clostridium innocuum* in HS, there was evidence that the IBD (inflammatory bowel disease) group had an excessive number of *Clostridium innocuum* colonization (Yu et al., 2023). *Clostridium innocuum* group had been postulated as a potential causative relation with Crohn’s disease (CD) by leading to creeping fat, stimulating tissue remodeling and causing inflamed and fibrotic intestine via M2 macrophages (Ha et al., 2020) which could partly explain the anti-protective mechanism the HS.

However, given the intricacy of the gut microbiota and the significant intra- and inter-species variation that might have an effect on host health, there was in fact a discrepancy between our findings and the available data. Conclusive evidence also needed to confirm how Lachnospira group increased the risk of IDB because Lachnospira group as one of butyrate-producing fora could benefit to certain inflammatory disorder (Wright et al., 2017; Iavarone et al., 2023; Ordoñez-Rodriguez et al., 2023). On the other hand, Lachnospira eligens, previously were known as *Eubacterium eligens* (Oren and Garrity, 2020). There was documentation of the evidence against the Eubacterium group which suppressed CD83 to preserve mice in systemic inflammation, corresponding to research by Islam et al. (2021). The Eubacterium group could contribute a proinflammatory function in colon carcinoma which could be similarly relevant to the role of nucleotide-binding oligomerization domain 2 linking HS with IBD, as demonstrated by Wang et al. (2021), Mintoff et al. (2023), and Ring et al. (2023). We thus concluded that the Lachnospira group might worsen HS by triggering systemic inflammation in the dysbacteriosis environment. Meanwhile, additional investigation was required done to determine this precise process. Research on the gut microbiota’s function in hidradenitis suppurativa is still under progress. The fundamental connection between the gut microbiota and hidradenitis suppurativa was based on the casual relation between gut microbiota and IBD (Chen and Chi, 2019). Moreover, anti-TNFα medications were effective in treating both HS and IBD, suggesting comparable inflammatory pathomechanisms. A potential new field of study involved employing bacteria to influence the immune system in therapeutics, since our awareness of the gut microbiome’s function in disease is expanding.

Our research revealed a link between four gut bacteria genera and HS. But HS is a multifactorial illness that may be impacted by the environment, gender, lifestyle, food, age, epigenetics, and genetics (Nomura, 2020). A previous study found that people with HS had considerably lower microbial makeup (α-diversity), and that *Ruminococcus gnavus* and *Clostridium ramosum* levels were higher in their microbiota than in those of healthy controls (McCarthy et al., 2022). Additionally, in 2020, Kam et al. analyzed fecal samples from three individuals with Hurley stage II or III in a case series. According to the study, gut microbiota species diversity might have diminished, there might be a rise in the number of Bilophila and Holdemania, a drop in the number of protective Lachnobacterium and Veillonella, and HS might be related to these changes. Additionally, it was observed that the phylum Firmicutes was decreased in the HS group compared to controls. But the authors pointed out that studies had shown that smoking can lower the relative abundance of Firmicutes in the gut (Kam et al., 2021). Lam and colleagues discovered that none of the healthy controls and the majority of HS patients had Robinsoniella in their feces, which might be properly quantified in the further inquiry (Lam et al., 2021). The occurrence of HS could not be explained by a change in a single bacterium, although a particular species might have a different sort of effect in a particular biological setting which demonstrated that a single bacterium would not have an impact on HS susceptibility. Hence, the discrepancy between our results and certain other studies might have the explanation.

As far as we are aware, this is the first MR research to examine the genetic relationship between HS and the gut flora. This study’s primary strength was its use of MR analysis to reduce confounding-related bias, which increased its trustworthiness when compared to traditional observational research. Through the targeting of certain gut flora, these discoveries provided new insights into the prevention, development, and therapy of HS. To investigate the precise processes behind the relationships between the gut microbiota and HS, further clinical trials and mechanism studies are required in the future.

However, our study has a number of drawbacks. First off, the majority of individuals in the two GWASs were of European heritage, so further research is needed to determine whether our findings are applicable. Second, the present microbiome GWAS research methodologies constrained the depth of our investigation. The generalizability of our results and the precision of our study may both be enhanced by adopting an advanced analytical approach to boost the specificity and accuracy of the existing results. What’s more, in this investigation, gender was not restricted. Hence, it was imperative to examine if there was a distinction between the male and female populations exclusively. By merging the information from cohort studies, clinical trials, and functional investigations, further work must be done to find connections between HS and gut microbiota which will be helpful for examining the pathophysiology of HS.

## 5 Conclusion

With final analysis, our bi-directional MR analysis revealed evidence of a putative causal link between certain gut microbiota and HS. Our findings would strengthen the case for gut microecological therapy of HS and establish a strong framework for more research into the pathophysiology of the gut microbiota that causes HS.

## Data availability statement

The original contributions presented in the study are included in the article/Supplementary material, further inquiries can be directed to the corresponding author.

## Author contributions

CL: Writing – original draft, Writing – review & editing. XCL: Data curation, Writing – review & editing. XL: Funding acquisition, Writing – review & editing.

## Footnotes

^1^https://mibiogen.gcc.rug.nl/, Accessed on March 12, 2023

^2^https://finngen.gitbook.io/documentation/, Accessed on March 12, 2023
